# Nomophobia and Its Association with Depression, Anxiety and Stress (DASS Scale), among Young Adults in Greece

**DOI:** 10.3390/ejihpe13120191

**Published:** 2023-11-26

**Authors:** Charalambos Gnardellis, Elissavet Vagka, Areti Lagiou, Venetia Notara

**Affiliations:** 1 Department of Fisheries and Aquaculture, School of Agricultural Sciences, University of Patras, 30200 Messolonghi, Greece; 2 Department of Public and Community Health, School of Public Health, University of West Attica, 12243 Athens, Greece; elvagka@uniwa.gr (E.V.); alagiou@uniwa.gr (A.L.); vnotara@uniwa.gr (V.N.)

**Keywords:** nomophobia, depression, anxiety, stress, self-esteem, smartphone overuse

## Abstract

Smartphones with their numerous applications have become essential daily equipment, prompting scientific research to deal with the impact of their use on psychosocial health. Under this spectrum, the aim of the present cross-sectional study was to examine the association between nomophobia and the negative emotional states of depression, anxiety, and stress, in relation to self-esteem and sociodemographic data, among the young adult population. The study sample consisted of 1408 young adults aged 18–25 years, participating on a voluntary basis with an online anonymous questionnaire. Data were collected through the “Nomophobia Questionnaire (NMP-Q)”, “Depression Anxiety Stress Scales—short form (DASS-21)”, and Rosenberg Self-Esteem Scale (RSES). The questionnaire also included socio-demographic characteristics and smartphone use variables. Data analysis showed that women were identified with severe depression and stress to a greater extent than men (63.3% vs. 55.1% for depression and 18.1% vs. 13.8% for stress scale). With respect to nomophobia, participants with severe levels of nomophobia also exhibited severe levels of negative emotional states in all DASS components, i.e., 40.6% in depression, 73.7% in anxiety, and 32.7% in stress (all *p* values < 0.001). Participants with severe levels of depression and anxiety were very often checking their phone and used it in all daily activities. Moreover, correlation analysis revealed that self-esteem had a moderating effect on the relationship between nomophobia and DASS, a fact that modifies the association between the involved variables: stronger relationships appeared between nomophobia and DASS components in individuals with normal/high self-esteem than in individuals with low self-esteem.

## 1. Introduction

Smartphones have become an inseparable element of contemporary life [1], causing behavioral changes in individuals’ daily routines and manners [2]. Nowadays, the development of smartphone technology provides great opportunities and conveniences for people [3]. As a result of their communication capabilities and people’s interaction with them, they have gained widespread acceptance [4]. According to users, smartphones have become an extension of their body, defining their identity and manner of being [5].

Depression is a widespread and significant disorder, affecting mood, thinking, and behavior. It is characterized by emotions of melancholy and/or a decline in appreciating interests. It can result in a range of mental and physical complications, impairing capacity in several activities [6]. Anxiety is a negative emotional condition characterized by tension feelings, concerned thoughts, and physical symptoms, such as tachycardia and sweating. Anxious individuals may react fearfully and dreadfully to particular issues and circumstances [7]. Finally, stress may be delineated as a condition characterized by apprehension or psychological strain arising from challenging circumstances. It represents a natural, innate human reaction that motivates confrontation and management of the difficulties and potential threats. However, long-term stressful situations are associated with mental health disorders, with anxiety and depression being the most prevalent outcomes. Nevertheless, the way in which people react to stress plays a significant role in determining their psychosocial well-being [8].

Nomophobia (derived from the term “no-mobile-phone-phobia”) is the dread of being separated from or unable to access one’s mobile phone. Nomophobia has the potential to transition into an addictive pattern, constituting a prevalent issue affecting young adults globally [9]. Moreover, individuals might feel depression [10], anxiety, stress, discomfort, and nervousness as a result of being unable to contact smartphone services [11], and, therefore, numerous consequences can manifest across negative emotional states [12]. Recent studies stated that an association exists between nomophobia and the presence of depression, anxiety, and stress [13,14].

Furthermore, scientific findings indicate that the excessive use of smartphones have psychopathological effects such as addiction, obesity, anxiety, depression, aggression, personality disorders, and loneliness [15,16,17]. Additionally, the stress and distraction caused by excessive smartphone use has a negative effect on academic learning and behavior, as well as on students’ wellbeing [18,19,20]. Moreover, the link between nomophobia and smartphone use, smartphone addiction, and internet addiction was found to be positive in terms of social anxiety and social media use [21,22].

There is a lack of studies that have investigated the relationship between nomophobia and negative emotional states such as anxiety, stress, and depression. Individuals with such issues were found to be more prone to exhibit nomophobic behaviors [23,24]. Examining whether a relationship exists between nomophobia and specific negative psychological states, including anxiety, depression, distress, insomnia, and impulsivity, would constitute a valuable and reinforcing contribution to the existing initiatives aimed at prevention and intervention. As technology continues to exert a substantial influence over various aspects of individuals’ lives, nomophobia could potentially evolve into a prevalent issue wherein individuals are physically present but psychologically disengaged. The potential adverse impact of nomophobia on mental well-being underscores the significant importance of investigating this phenomenon comprehensively to establish preventive strategies and potential therapeutic interventions [25].

Despite the extensive use of mobile phones in Greece, there are limited scientific studies dealing with the emerging phenomenon of nomophobia in the country [26,27]. Hence, the purpose of the present investigation was to examine the association between nomophobia and the negative emotional states of depression, anxiety, and stress among young Greek adults. The investigated hypotheses were examined without defining an a priori causal direction for the involved variables. It was defined in terms of correlation coefficients, avoiding the investigation of a specific causal model, which would assume an initial condition and a probable outcome. Moreover it was taking into account the possible confounding effect of the socio-demographic data and the self-esteem variable in order to test whether they modify or mediate the examined relationships.

## 2. Materials and Methods

### 2.1. Procedure

The data collection process entailed the distribution of a self-administrated questionnaire during lectures throughout the 2020–21 academic year. In light of the COVID-19 restrictions, the study’s lead researcher made all requisite information available online and it was accessible via the Microsoft Teams platform during the questionnaire’s completion. Consequently, data were acquired electronically.

The study received approval from the research committee of the University of West Attica (Approval Number: 14/21-09-2020) and adhered to the ethical principles outlined in the Declaration of Helsinki (1964) and its subsequent revisions. Participants were provided with comprehensive information about the study’s purpose and methodology, and their voluntary consent was obtained prior to their participation.

### 2.2. Participants

The present cross-sectional study encompassed a sample of 1408 male and female students aged between 18 and 25 years. The participants were recruited from six different faculties within the University of West Attica and Post-Secondary Vocational Training institutions situated in Athens, the capital city of Greece. It should be mentioned that the University is the third largest in Greece regarding the number of students. The selection of participants adhered to predefined inclusion criteria, such as (a) possession of a smartphone, (b) age range of 18 to 25 years, and (c) completion of the informed consent form.

### 2.3. Measures

The questionnaire was structured into five distinct sections, encompassing the following domains: (a) socio-demographic characteristics, which encompassed factors such as age, gender, education level, place of residence, and parents’ educational background; (b) smartphone usage patterns, including metrics such as daily hours of use, the purpose of using a smartphone, and frequency of calls and text messages; (c) the nomophobia questionnaire (NMP-Q); (d) the Depression Anxiety Stress Scale-21 (DASS-21); and (e) the Rosenberg Self-Esteem Scale (RSES).

#### 2.3.1. Nomophobia Questionnaire (NMP-Q)

The Nomophobia Questionnaire (NMP-Q) is a 20-item Likert scale that utilizes a 7-point continuum ranging from 1 denoting ‘strongly disagree’ to 7 signifying ‘strongly agree.’ Calculation of NMP-Q scores involves summing up the responses, yielding a numerical value within the range of 20 to 140. The maximum score (NMP-Q = 140) reflects the most severe manifestation of nomophobia. Conversely, a score of 20 indicates the absence of nomophobia, while scores falling within the range of 21 to 59 denote mild nomophobia, scores from 60 to 99 signify moderate nomophobia, and scores ranging from 100 to 140 indicate severe nomophobia. Furthermore, the NMP-Q encompasses four distinct dimensions, namely: (a) Not being able to communicate, (b) Losing connectedness, (c) Not being able to access information, and (d) Giving up convenience.

The original iteration of the NMP-Questionnaire was formulated by Yildirim and Correia (2015) [28], subsequently undergoing validation for use in the Greek language. Both exploratory and confirmatory factor analyses conducted on the Greek version revealed a four-factor structure (subscales) consistent with the original instrument. Additionally, an overall nomophobia scale was derived from the amalgamation of all NMP-Q items [29]. The total scale exhibited robust internal consistency, as evidenced by Cronbach’s alpha coefficients of 0.945 for both versions of the questionnaire. Furthermore, the Cronbach’s alpha values for each individual factor were as follows: (a) 0.936, (b) 0.895, (c) 0.867, and (d) 0.854, closely approximating those observed in the original NMP-Q, which were 0.939, 0.827, 0.819, and 0.874, respectively.

#### 2.3.2. Depression Anxiety Stress Scale-21 (DASS-21)

The DASS-21 (Depression Anxiety Stress Scale-21) represents a concise version of Lovibond and Lovibond’s (1995) questionnaire [30], which originally comprised 42 items. This abbreviated instrument is structured into three distinct subscales, each containing seven items, aimed at assessing depression (Depression scale), anxiety (Anxiety scale), and stress (Stress scale). Within the depression scale, symptoms such as distress, hopelessness, diminished interest/engagement, and anhedonia are evaluated (specifically, questions 3, 5, 10, 13, 16, 17, and 21). The anxiety scale focuses on aspects including autonomic nervous system arousal, musculoskeletal system effects, state anxiety, and subjective experiences related to anxiety (specifically, questions 2, 4, 7, 9, 15, 19, and 20). Meanwhile, the stress scale is designed to gauge a state of chronic arousal and tension characterized by difficulties in relaxation, overstimulation, heightened aggression, irritability, and impatience (specifically, questions 1, 6, 8, 11, 12, 14, and 18). Participants are asked to rate the severity of each symptom they experienced during the preceding week using a 4-point Likert-type scale (0 = not applicable at all, 3 = very applicable or most of the time), with scores ranging from 0 to 3 points for each statement. To derive the total negative affective state score for each participant, the scores for all items are summed. To obtain the final score on the DASS-21 questionnaire, this total score is then multiplied by 2. The maximum achievable score for the entire questionnaire is 63, and, for each subscale, it is 21. Depression scores are categorized as follows: normal (0–9), mild (10–13), moderate (14–20), severe (21–27), and extremely severe (28 and above). Anxiety scores are classified as normal (0–7), mild (8–9), moderate (10–14), severe (15–19), and extremely severe (20 and above). Stress scores fall into the following categories: normal (0–14), mild (15–18), moderate (19–25), severe (26–33), and extremely severe (34 and above).

The Cronbach’s alphas of the study DASS components were 0.90, 0.88, and 0.88 for depression, anxiety, and stress, respectively, suggesting a high internal consistency of the DASS scales. In the Greek context, the 42-item scale has been validated by Lyrakos et al. (2011) [31], with a reported Cronbach’s alpha of 0.965, and the 21-item DASS scale has been validated by Pezirkianidis et al. (2018) [32], yielding Cronbach’s alpha coefficients ranging from 0.84 to 0.85 for all scale items.

#### 2.3.3. Rosenberg Self-Esteem Scale (RSES)

The RSES (Rosenberg Self-Esteem Scale) is a questionnaire consisting of ten items designed to evaluate an individual’s overall self-esteem by capturing both positive and negative self-perceptions. This scale is widely acknowledged for its reliability and accuracy in assessing one’s self-worth quantitatively. Responses are recorded on a Likert scale ranging from 0 to 3, where 0 signifies “strongly disagree”, and 3 signifies “strongly agree”. Notably, items 3, 5, 8, 9, and 10 are reverse scored. The total score is calculated by summing the Likert values, with a cumulative score below 15 indicating low self-esteem, a score within the range of 15 to 25 indicating a normal level of self-esteem, and a score exceeding 25 signifying high self-esteem. Originally designed to assess self-esteem among high school students, this scale has since been utilized across various age groups (i.e., adults) [33]. The study’s Cronbach’s alpha coefficient for the RSES was calculated at 0.81, indicating a high degree of internal consistency, a finding consistent with previous research by Galanou et al. in 2014 [34].

### 2.4. Data Analysis and Statistical Methods

Data analysis was undertaken by simple univariate techniques and correlation analysis. Nominal and ordinal variables were presented as absolute and relative (%) frequencies. The associations between nomophobia levels, sociodemographic characteristics of participants, and DASS components were evaluated through χ^2^ for linearity. Central tendency of continuous variables was given by their mean values, while comparisons between them, due to rejection of equal variance assumption, were evaluated through the Brown-Forsyth analysis of variance. Internal consistency of nomophobia and DASS scales was evaluated by means of Cronbach’s alpha. For the sake of a more coherent description of the results, the categories of the DASS scales were merged into 3 for each emotional state: normal, mild/moderate = moderate, and severe/extremely severe = severe.

Pearson’s correlation coefficients were estimated for the relationships between the nomophobia total score and DASS components: depression, anxiety, stress, and total DASS scale. In order to control for possible confounding effects of basic sociodemographic characteristics, partial correlation coefficients between nomophobia and DASS scales were calculated, controlling for gender, age, working status, education, residency, nationality, and parents’ educational level. Due to the interactive role of self-esteem on the nomophobia and DASS relationship, all the above correlations were estimated separately for the subjects with low and high/normal self-esteem.

Simple and partial correlation coefficients are given, along with their corresponding 95% confidence intervals (95% CI). In the first run, the simple correlation coefficients between nomophobia and DASS components were estimated. Then, they were adjusted for participants’ sociodemographic variables and the moderating effect of self-esteem. All statistical assessments were performed using SPSS v. 28 statistical software (IBM Corp, Armonk, NY, USA).

## 3. Results

From the total sample, the majority were women (71.7%), while the age groups (18–20, 21+) were equally distributed. More than half (57.0%) of the participants exhibited moderate level of nomophobia. Most of the participants were living with their parents (74.2%), while only a relatively small percentage of all participants identified with low levels of self-esteem (18.5%). Regarding the sociodemographic characteristics in relation to DASS scales, it was observed that women, compared with men, exhibited higher rates of severe anxiety (63.3% vs. 55.1%) and stress (18.1% vs. 13.8%). Additionally, severe levels of depression, anxiety, and stress prevailed among participants with low levels of self-esteem (72.0%, 88.5%, and 36.8%, respectively). With respect to nomophobia, participants with severe levels of nomophobia also exhibited severe levels in all components of the DASS scale (40.6% in depression, 73.7% in anxiety, and 32.7% in stress) (Table 1).

Table 2 shows the results of correlation analysis between nomophobia and DASS. Pearson’s correlation coefficients were estimated between the nomophobia total score and DASS components: depression, anxiety, stress, and total DASS score. In a second run, partial correlation coefficients between nomophobia and DASS scales were also estimated, controlling for gender, age, working status, education, residency, nationality, and parents’ education. Due to the interactive role of self-esteem on the nomophobia and DASS relationship, all the above correlations were estimated separately for the participants with low and high/normal self-esteem.

The estimated partial correlation coefficients, after controlling for participants’ sociodemographic data, did not differ substantially from the simple ones, implying that control variables did not confound the relationship between nomophobia and DASS. In participants with high self-esteem, the partial correlation coefficients between the total nomophobia score and the DASS parameters were in the range of 0.24–0.28, apart from depression, whose correlation coefficient was quite lower (*r* = 0.16). On the contrary, in people with low self-esteem, the corresponding partial correlation coefficients were much lower (0.18 for stress and total DASS, 0.19 for anxiety), while for depression the relationship did no longer exist (*r* = 0.09, *p* value = 0.141) (Table 2).

Since nomophobia is highly associated with the extensive use of smartphones, a further analysis was conducted to clarify the association in detail. It was observed that participants with severe levels of depression, anxiety, and stress were very often checking their phone, i.e., up to 10 min (39.4%, 39.7%, and 44.1%, respectively). On a daily basis, participants identified as severely depressed were using their phone for playing games more often than others (51.7% vs. 43.3% for moderate depressed and 41.1% for normal), while their use to communicate with family/friends was to a lesser extent compared with the rest (95.1% vs. 97.5%). Regarding the circumstances under which they used their phones, participants with severe depression used them to a greater degree during eating (42.1%), during lessons (47.5%), while watching TV (62.8%), and while they were with friends (35.7%) (all *p* values < 0.05). Participants with severe anxiety also used their phones more often than others during eating (39.5%) and during lessons (45.1%) (*p* values are 0.022 and 0.003, respectively). Regarding severely stressed participants, they used their phones less to communicate with their friends and more for using the camera (*p* values 0.023 and 0.019, respectively), while their use increased compared with the other participants during eating (47%), when they were with friends (37.4%) and while watching TV (63.4%) (all *p* values ≤ 0.05) (Table 3).

Regarding the relationship between internet-based social communication and DASS components, it was observed that participants identified with a severe level in all DASS components used the phone more hours per day (i.e., almost 7 h/day) compared with those with normal and moderate levels (all *p* values < 0.01). Those with severe levels of stress had more followers (746 vs. 631 and 526, respectively) and exchanged more emails/day (8.2 vs. 7.4 and 7.6, respectively) compared with those with normal and moderate levels (all *p* values < 0.05). Participants identified with severe depression used their smartphones more hours/day, even though they made less calls/day compared with those with normal and moderate levels (5.7 vs. 7 and 7.3, respectively, *p* value < 0.003) (Table 4).

## 4. Discussion

The widespread integration of smartphones into individuals’ daily life has led to the increased prevalence of their use and the appearance of the phenomenon known as “nomophobia”. Nowadays, while smartphones have undoubtedly facilitated life, the harmful consequences stemming from their problematic utilization have been on a swift and upward trajectory. Furthermore, their excessive use has been linked to the development of a range of negative emotional conditions, such as depression, anxiety, stress, and low self-esteem [13,14,35].

In the present study, the majority of participants (57%) demonstrated a moderate level of nomophobia. This aligns with findings from previous studies, indicating that individuals displayed a moderate level of nomophobia [23,36,37,38]. It was also noted that a relatively small percentage of all participants identified with low levels of self-esteem (18.5%), which is similar to other studies among university students [39].

Concerning the DASS components, it was observed that females, compared with males, revealed more severe levels of anxiety (63.3% vs. 55.1%) and stress (18.1% vs. 13.8%). Santl, Brajkovic and Kopilaš (2022) [40,41] reported that gender differences do exist as regards the manifestation of anxiety and stress. Furthermore, severe levels of depression, anxiety, and stress prevailed among participants with low levels of self-esteem (72.0%, 88.5%, and 36.8%, respectively). It is known from the literature that low self-esteem is associated with the DASS mental states. These relationships are usually defined as bidirectional, in the sense that low self-esteem can increase depression, anxiety, and stress, and these in turn have the opposite effect by further eroding self-esteem. Results from several empirical studies highlight this relationship, documenting it for all three DASS scales [42,43,44,45,46,47].

Describing in general terms the relationship of nomophobia with DASS negative states, we would say that participants with severe levels of nomophobia revealed severe levels in all components of DASS (40.6% in depression, 73.7% in anxiety, and 32.7% in stress, respectively). Nomophobia is a prevalent and emerging issue, predominantly in younger individuals, and it is closely associated with symptoms of depression, anxiety, and stress [40,48,49,50]. From the correlation analysis, positive correlation coefficients were determined between nomophobia and the depression, anxiety, and stress scales. These relationships are quite strong in individuals with normal or high self-esteem for the anxiety and stress scales (0.24 and 0.28, respectively) and secondarily for that of depression (0.19). In individuals with low self-esteem, the positive correlations appear much lower for the anxiety and stress scales (0.18 and 0.19, respectively), while they disappear in the case of depression. One possible interpretation is that low self-esteem is already a strong risk factor for high scores in depression, anxiety, and stress scales, as already mentioned, so that the mediating scope of nomophobia to further increase the scales is limited. In contrast to people with normal self-esteem, the presence of negative states of depression, anxiety, and stress, due to the lack of a pre-existing background attributed to low self-esteem, may arise to a greater extent due to nomophobia and excessive use of the mobile phone [51,52].

Concerning smartphone use, it was observed that individuals exhibiting severe levels of depression and anxiety were very often checking their phones (usually up to 10 min). They were more prone to use them for gaming, while their use for communication with family and friends was comparatively less frequent than the others. As it appears from the literature, individuals with smartphone overuse exhibit higher levels of depression [53], while a more proper use was associated with lower levels of depression, anxiety, and stress among college students, which implies a close relationship between smartphone use and mental well-being [54,55].

Regarding daily activities during which participants used their phones, those with severe depression, anxiety, and stress reported smartphone overuse during eating, lectures, while watching TV, and when socializing with friends. Moreover, individuals identified with severe stress used their smartphones less to communicate with their friends and more for using the camera. In the same direction, the results of another study revealed that the majority of participants used their smartphones in various daily situations (i.e., during meals, lectures, while driving, socializing with friends, etc.) depending also on the severity of depression, stress, and anxiety levels [24].

Concerning the link between internet-based social communication and DASS components, individuals identified with severe depression, anxiety, and stress used smartphones for longer daily durations (approximately 7 h) compared with those with normal and moderate levels. Severe stress was associated with more followers and email exchanges. Severe depression, despite increased smartphone use, resulted in fewer calls per day. A study conducted in Jordan showed that a significant percentage of students suffered from a severe form of depression (23.7%), anxiety (22.6%), and stress (15.4%), and primarily used social networking sites for entertainment purposes, such as viewing movies and sharing humorous content like jokes or images. Notably, this recreational use was significantly correlated with symptoms of psychological distress [56].

In this part, as regards the methodological point of view of the study, it should be mentioned that the relationship between nomophobia and the negative emotional states of depression, anxiety, and stress without defining a hypothesized direction of this relationship was examined. It was defined in terms of correlation coefficients, avoiding the investigation of a specific causal model, which would assume an initial condition and a probable outcome (which would allow us to measure the risk of that outcome in stochastic terms, e.g., through the relative risk or the odds ratios). It could be considered, for example, that strongly nomophobic individuals end up showing more frequent severe symptoms of depression, anxiety, or stress. Or, conversely, that depressed, anxious, or stressed identified individuals tend to be more dependent on their mobile devices as a result, possibly, of their pathology. However, in most of the papers that have been published on the relationship between nomophobia and DASS components, the direction of cause and effect has not been defined in advance, and usually this relationship is estimated through correlation coefficients [49,57,58]. And this is because its interpretation can hardly be attributed to a causal scheme of cause and effect, due to the potential bidirectional relationship that nomophobia can have with the DASS components. In a more complex interpretation of the relationships, we could hypothesize that nomophobic individuals have a greater risk of depression, anxiety, or stress, which in turn would exacerbate the dependence on their mobile device or the fear of not having access to it. Or, conversely, that people identified as depressed (or anxious or stressed) are more likely to have nomophobia, which further exacerbates their depression (or anxiety, stress).

However, the stochastic models that could test the previous two cases are quite complex and with high uncertainty to avoid biases and type I errors. The investigation might have been possible through structural equation modeling (SEM) if exogenous variables were included in the analysis that were related to known relationships only with nomophobia variables or only with DASS variables (something that does not exist in our data set) [59]. In any case, however, the cross-sectional nature of the present study makes it very difficult to investigate bidirectional causal relationships between nomophobia and DASS. A longitudinally designed epidemiological study with these as key variables could illuminate the problem in more detail.

### Limitations

This is a cross-sectional study and it is not possible to establish causal relationships based on the findings. Moreover, these findings were derived from a sample of young adult students, with a higher representation of women than men. Additionally, the use of self-report measures may introduce response and recall biases. It is crucial to highlight that the current study focuses on self-reported symptoms rather than real mental disorders or clinical diagnoses. Nonetheless, the findings provide valuable insights into the specific issue.

## 5. Conclusions

In the present study, a close relationship was observed between nomophobic behaviors and levels of depression, anxiety, and stress. Healthcare professionals should be aware about issues regarding nomophobia and its relation to negative emotional symptoms. Interventions targeting on nomophobia may offer additional benefits in the improvement of anxiety and stress, by providing guidance on responsible smartphone use and healthy digital behaviors. Further research can highlight the hazards of nomophobia and its association with depression, anxiety, and stress. It must be emphasized that the safe use of smartphones should be integrated into health education and health promotion programs, in order to protect young people’s well-being. Due to the potential bidirectional relationship between nomophobia and DASS components, caution is essential in the implementation of interventions, so as to enable young adults to confront these emerging issues effectively.

## Figures and Tables

**Table 1:** Sociodemographic characteristics, NMP and self-esteem levels by DASS components ^1^.

			Depression				Anxiety				Stress			
	N	%	Normal	Moderate	Severe		Normal	Moderate	Severe		Normal	Moderate	Severe	
			(559)	(443)	(406)	*p* Value *	(254)	(295)	(859)	*p* Value *	(911)	(259)	(238)	*p* Value *
			39.7	31.5	28.8		18.0	21.0	61.0		64.7	18.4	16.9	
Gender														
Women	(1009)	71.7	39.8	30.4	29.7	0.329	17.2	19.4	63.3	0.017	62.2	19.6	18.1	0.004
Men	(399)	28.3	39.3	34.1	26.6		20.1	24.8	55.1		70.9	15.3	13.8	
Age groups														
18–20	(697)	49.5	36.7	32.3	30.9	0.019	17.0	20.8	62.2	0.289	64.3	18.6	17.2	0.736
21+	(711)	50.5	42.8	30.6	26.7		19.1	21.1	59.8		65.1	18.2	16.6	
Work														
No	(964)	68.5	37.6	32.1	30.4	0.013	17.8	19.7	62.4	0.245	64.1	18.8	17.1	0.561
Yes	(444)	31.5	44.4	30.2	25.5		18.5	23.6	57.9		66.0	17.6	16.4	
Education														
University	(1060)	75.3	38.3	32.3	29.4	0.111	17.6	20.6	61.8	0.320	65.1	19.1	15.8	0.217
Post-secondary	(348)	24.7	44.0	29.0	27.0		19.3	22.1	58.6		63.5	16.4	20.1	
Residency														
With parents	(1045)	74.2	38.2	32.2	29.6	0.080	17.6	20.6	61.8	0.310	65.2	18.2	16.7	0.551
Alone	(363)	25.8	44.1	29.2	26.7		19.3	22.0	58.7		63.4	19.0	17.6	
Nationality														
Greek	(1319)	93.9	39.8	31.5	28.7	0.758	17.7	21.3	61.0	0.815	65.2	18.0	16.8	0.212
Other	(85)	6.1	38.8	30.6	30.6		22.4	14.1	63.5		56.5	24.7	18.8	
Nomophobia														
Mild	(339)	24.1	52.8	26.0	21.2	<0.001	30.4	23.9	45.7	<0.001	76.4	14.7	8.8	<0.001
Moderate	(803)	57.0	37.2	34.6	28.1		15.2	21.5	63.3		65.8	19.2	15.1	
Severe	(266)	18.9	30.5	28.9	40.6		10.9	15.4	73.7		46.6	20.7	32.7	
Self-esteem														
Low	(261)	18.5	4.2	23.8	72.0	<0.001	4.2	7.3	88.5	<0.001	33.3	29.9	36.8	<0.001
Normal/High	(1147)	81.5	47.8	33.2	19.0		21.2	24.1	54.8		71.8	15.8	12.4	

^1^ Normal, Moderate = mild/moderate, Severe = severe/extremely severe. * *χ*^2^ for linear trend.

**Table 2:** Pearson and partial correlation coefficients between NMPQ score and DASS components by self-esteem categories.

Low Self-Esteem	Pearson correlation coefficients between NMPQ Score and DASS components											
	Depression			Anxiety			Stress			DASS		
	*r*	95% CI	*p* value	*r*	95% CI	*p* value	*r*	95% CI	*p* value	*r*	95% CI	*p* value
NMPQ score	0.10	−0.02–0.22	0.094	**0.22**	0.10–0.33	**<0.001**	**0.18**	0.06–0.30	**0.003**	**0.20**	0.08–0.31	**0.001**
	Partial correlation coefficients ^1^ between NMPQ score and DASS components											
	Depression			Anxiety			Stress			DASS		
	*r*	95% CI	*p* value	*r*	95% CI	*p* value	*r*	95% CI	*p* value	*r*	95% CI	*p* value
NMPQ score	0.09	−0.03–021	0.141	**0.19**	0.07–0.31	**0.002**	**0.18**	0.05–029	**0.005**	**0.18**	0.06–030	**0.003**
Normal/High Self-esteem	Pearson correlation coefficients between NMPQ score and DASS components											
	Depression			Anxiety			Stress			DASS		
	*r*	95% CI	*p* value	*r*	95% CI	*p* value	*r*	95% CI	*p* value	*r*	95% CI	*p* value
NMPQ score	**0.17**	0.11–0.23	**<0.001**	**0.25**	0.20–0.30	**<0.001**	**0.29**	0.23–0.34	**<0.001**	**0.27**	0.21–0.32	**<0.001**
	Partial correlation coefficients ^1^ between NMPQ score and DASS components											
	Depression			Anxiety			Stress			DASS		
	*r*	95% CI	*p* value	*r*	95% CI	*p* value	*r*	95% CI	*p* value	*r*	95% CI	*p* value
NMPQ score	**0.16**	0.10–021	**<0.001**	**0.24**	0.18–0.29	**<0.001**	**0.28**	0.22–032	**<0.001**	**0.25**	0.19–030	**<0.001**

^1^ Controlling for gender, age, work status, education, residency, nationality, and parents’ education. All *p* values are referred to *t*-test for correlation coefficients.

**Table 3:** Smartphone use by DASS components. Percentages of use by Depression, Anxiety, and Stress categories *.

		Depression				Anxiety				Stress			
	Total	Normal	Moderate	Severe	*p* Value ^1^	Normal	Moderate	Severe	*p* Value ^1^	Normal	Moderate	Severe	*p* Value ^1^
Checking													
Up to 10 min	36.1	30.1	40.6	39.4	<0.001	26.4	33.9	39.7	<0.001	33.0	39.4	44.1	<0.001
20 min	18.8	16.1	19.2	21.9		13.4	18.6	20.4		17.0	21.2	22.7	
30 min	16.8	21.1	12.9	15.3		22.4	17.3	15.0		18.8	14.7	11.8	
>30 min	28.3	32.7	27.3	23.4		37.8	30.2	24.9		31.2	24.7	21.4	
Smartphone use for Communication with family/friends	96.8	97.5	97.5	95.1	0.044	97.2	97.6	96.4	0.364	97.4	97.3	94.1	0.023
Social media	81.3	82.5	81.7	79.1	0.192	78.3	82.4	81.7	0.316	81.4	82.2	79.4	0.582
Games	44.9	41.1	43.2	51.7	0.001	41.3	41.7	47.0	0.059	43.5	46.3	48.7	0.123
Camera	72.5	71.4	76.7	69.5	0.660	71.3	70.8	73.5	0.387	70.5	75.3	77.3	0.019
Web-based information	90.8	91.2	92.6	88.4	0.176	90.2	91.5	90.8	0.865	90.6	92.3	90.3	0.872
Smartphone use													
During eating	37.2	34.2	36.3	42.1	0.015	32.4	34.4	39.5	0.022	34.4	37.8	47.0	<0.001
During lessons	42.2	37.6	43.1	47.5	0.002	35.0	40.0	45.1	0.003	40.7	44.0	45.8	0.123
During driving	2.8	2.7	2.3	3.4	0.522	3.9	2.0	2.7	0.433	2.6	1.2	5.0	0.160
With friends	32.2	29.0	33.0	35.7	0.025	29.1	30.2	33.8	0.118	30.2	34.4	37.4	0.023
In transportations	80.2	78.7	80.4	82.0	0.202	81.9	79.3	80.0	0.599	79.6	80.7	81.0	0.400
When watching TV	57.9	54.7	57.3	62.8	0.014	55.5	56.6	59.0	0.273	56.3	58.3	63.4	0.052

* Normal, Moderate = mild/moderate, Severe = severe/extremely severe. ^1^ *χ*^2^ for linear trend.

**Table 4:** Mobile phone use by DASS components. Mean values by Depression, Anxiety, and Stress categories *.

		Depression				Anxiety				Stress			
	Total Mean	Normal Mean	Moderate Mean	Severe Mean	*p* Value ^1^	Normal Mean	Moderate Mean	Severe Mean	*p* Value ^1^	Normal Mean	Moderate Mean	Severe Mean	*p* Value ^1^
Calls/day	6.7	7	7.3	5.7	0.003	7	6.4	6.8	0.624	6.5	6.9	7	0.696
Messages/day	24.7	23.9	25.4	25.2	0.435	22.5	25.6	25.1	0.083	25	22.9	25.7	0.217
Emails/day	7.6	7.5	7.6	7.7	0.599	7.5	7.3	7.7	0.299	7.4	7.6	8.2	0.016
Friends ^2^	1016	1077	1001	948	0.242	1060	998	1009	0.804	1017	913	1123	0.179
Followers ^3^	631	665	621	596	0.315	707	577	627	0.149	631	526	746	0.003
Phone use hours/day	6.7	6.4	6.6	7.3	<0.001	6.4	6.4	6.9	0.009	6.4	7.1	7.4	<0.001
Computer use hours/week	19.3	18.8	19.9	19.4	0.614	19.4	19.3	19.3	0.996	19.4	19.4	19	0.938

* Normal, Moderate = mild/moderate, Severe = severe/extremely severe. ^1^ Brown-Forsythe robust test of equality of means. ^2^ (Fb, MSN, games), ^3^ (Fb, Insta, Twitter).

## Data Availability

Data available on request due to restrictions eg privacy or ethical. The data presented in this study are available on request from the corresponding author.
